# Supplementary material for: Pathologic and molecular responses to neoadjuvant trastuzumab and/or lapatinib from a phase II randomized trial in HER2-positive breast cancer (TRIO-US B07)
Source: Nat Commun. 2020 Nov 17;11:5824. doi: 10.1038/s41467-020-19494-2 (PMC7673127; doi:10.1038/s41467-020-19494-2)
Supplement: Supplementary file 1 — Supplementary Information [file 41467_2020_19494_MOESM1_ESM.pdf]

Supplementary Tables and Figures

	Cohort with Baseline Samples (N=110)			Cohort with Baseline & Run-In Samples (N=89)			Cohort with Baseline & Surgical Samples (N=59)			Overall ITT Cohort (N=128)		
Arm N (%)	TCH	TCL	TCHL	TCH	TCL	TCHL	TCH	TCL	TCHL	TCH	TCL	TCHL
Total	31 (28)	29 (26)	50 (45)	26 (29)	25 (28)	38 (42)	14 (24)	14 (24)	31 (53)	34 (26)	36 (28)	58 (45)
HR+	17 (54)	15 (52)	32 (64)	13 (50)	12 (48)	24 (63)	7 (50)	8 (57)	21 (68)	20 (59)	18 (50)	34 (59)
HR-	14 (45)	14 (48)	18 (36)	13 (50)	13 (52)	14 (37)	7 (50)	6 (43)	10 (32)	14 (41)	18 (50)	24 (41)
pCR	15 (48)	7 (24)	25 (50)	12 (46)	7 (28)	19 (50)	6 (43)	4 (29)	15 (48)	16 (47)	9 (25)	30 (52)

Supplementary Table 1. Characteristics and outcomes for expression-evaluable cohorts and the overall cohort. HR: hormone receptor; pCR: pathologic complete response; T: docetaxel; C: carboplatin; H: trastuzumab; L: lapatinib.

	All Arms	Arm 1 (Trastuzumab)	Arm 2 (Lapatinib)	Arm 3 (Combination)	P-value for interaction*
**Hormone receptor status (N=110)**					0.70
HR-positive	0.344	0.412	0.133	0.406	
HR-negative	0.544	0.571	0.357	0.667	
OR (95% CI)	2.25 (1.04-4.98)	1.85 (0.435-8.38)	3.34 (0.55-30.6)	2.83 (0.86-10.24)	
**HER2 immunohistochemistry (N=73)**					
IHC 1+ or 2+	0.077	0	0	0.143	0.99
IHC 3+	0.533	0.60	0.313	0.621	
OR (95% CI)	11.9 (2.11 - 304.2)	NA	NA	8.40 (1.17-238.6)	
**Intrinsic subtype (N=110)**					
Not HER2-enriched	0.333	0.333	0.143	0.455	0.61
HER2-enriched	0.500	0.579	0.333	0.536	
OR (95% CI)	1.98 (0.91-4.41)	2.62 (0.59-13.47)	2.80 (0.46-25.5)	1.37 (0.44-4.35)	
**Integrative subtype (N=110)**					
Not iC5	0.250	0.167	0.20	0.308	0.46
iC5	0.477	0.560	0.25	0.568	
OR (95% CI)	2.67 (1.00-8.10)	5.51 (0.71-161.2)	1.22 (0.13-38.2)	2.84 (0.76-12.56)	
**Stromal TIL percentage (N=85)**					
10% or lower	0.403	0.421	0.294	0.462	0.25
Higher than 10%	0.609	0.750	0.286	0.750	
OR (95% CI)	2.27 (0.85-6.30)	3.62 (0.35-117.6)	0.99 (0.10-6.94)	3.31 (0.76-18.66)	
**On-treatment tumor (N=83)**					
Present	0.333	0.467	0.250	0.3125	
Absent	0.563	0.667	0.200	0.619	0.34
OR (95% CI)	2.53 (1.02-6.46)	2.14 (0.29-21.7)	0.81 (0.03-7.95)	3.41 (0.87-14.93)	
**HER2 FISH ratio (N=81)**	1.14 (1.01-1.30)	1.16 (0.94 - 1.58)	1.25 (0.93 - 1.79)	1.11 (0.93 - 1.36)	0.56
**Early estrogen signature (N=110)**	8.4E-6 (3.5E-9-8.7E-3)	6.9E-12 (1.7E-21-4.4E-5)	5.6E-4 (8.7E-14-7.4E4)	7.0E-5 (2.1E-9-0.64)	0.54
**GeparSixto immune signature (N=110)**	4.31 (0.77-25.6)	24.9 (0.63-1895.7)	0.63 (0.014-26.8)	6.46 (0.55-89.3)	0.20

Supplementary Table 2. Association of pre-treatment biomarkers with pathologic complete response (pCR) in the overall cohort and by intervention arm. For binary variables, proportion achieving pCR in each subgroup as well as odds ratio (OR) and 95% confidence interval (CI) are shown. For continuous variables, the odds ratio of pCR for every unit increase in the marker and 95% CI are shown. * P-values are for the two-sided Wald test of the coefficient of the interaction term in a logistic model predicting pCR from the variable of interest and the use of trastuzumab (Arms 1 and 3 vs Arm 2), without adjustment for multiple comparisons. IHC: immunohistochemistry; TIL = tumor-infiltrating lymphocyte; FISH = fluorescent in situ hybridization.

	4-variable model (N=81)	4v model + IHC (N=50)	4v model + IS (N=81)	4v model + IC (N=81)	4v model + on-treatment (N=62)
	OR (95% CI)	OR (95% CI)	OR (95% CI)	OR (95% CI)	OR (95% CI)
Nodal status (+ vs -)	3.67 (1.30-11.1)	2.23 (0.60-8.9)	3.37 (1.16-10.6)	3.72 (1.31-11.5)	2.68 (0.87-8.86)
Trastuzumab receipt (yes vs no)	6.60 (1.70-33.3)	4.72 (0.98-29.5)	6.44 (1.66-32.3)	6.81 (1.74-34.6)	4.58 (1.14-23.3)
HR-status (- vs +)	4.18 (1.42-13.6)	2.79 (0.68-13.4)	4.07 (1.34-13.1)	3.73 (1.23-12.4)	2.79 (0.87-9.73)
HER2 FISH (continuous)	1.21 (1.05-1.43)	0.99 (0.77-1.26)	1.20 (1.04-1.42)	1.19 (1.03-1.41)	1.16 (0.99-1.40)
HER2 IHC (3+ vs 1-2+)	--	7.92 (0.96-173.8)	--	--	--
Intrinsic subtype (HER2-enriched vs not)	--	--	1.39 (0.45-4.22)	--	--
Integrative subtype (iC5 vs not)	--	--	--	1.75 (0.45-7.29)	--
On-treatment tumor (absent vs present)	--	--	--	--	1.48 (0.45-4.90)

Supplementary Table 3. Association of biomarkers with pathologic complete response (pCR) in multivariate analysis. For HER2 FISH ratio (continuous), the odds ratio of pCR for every unit increase in the marker is shown. IHC = immunohistochemistry (HER2); IS = intrinsic subtype; IC = integrative cluster; OR = odds ratio; CI = confidence interval; HR = hormone receptor.

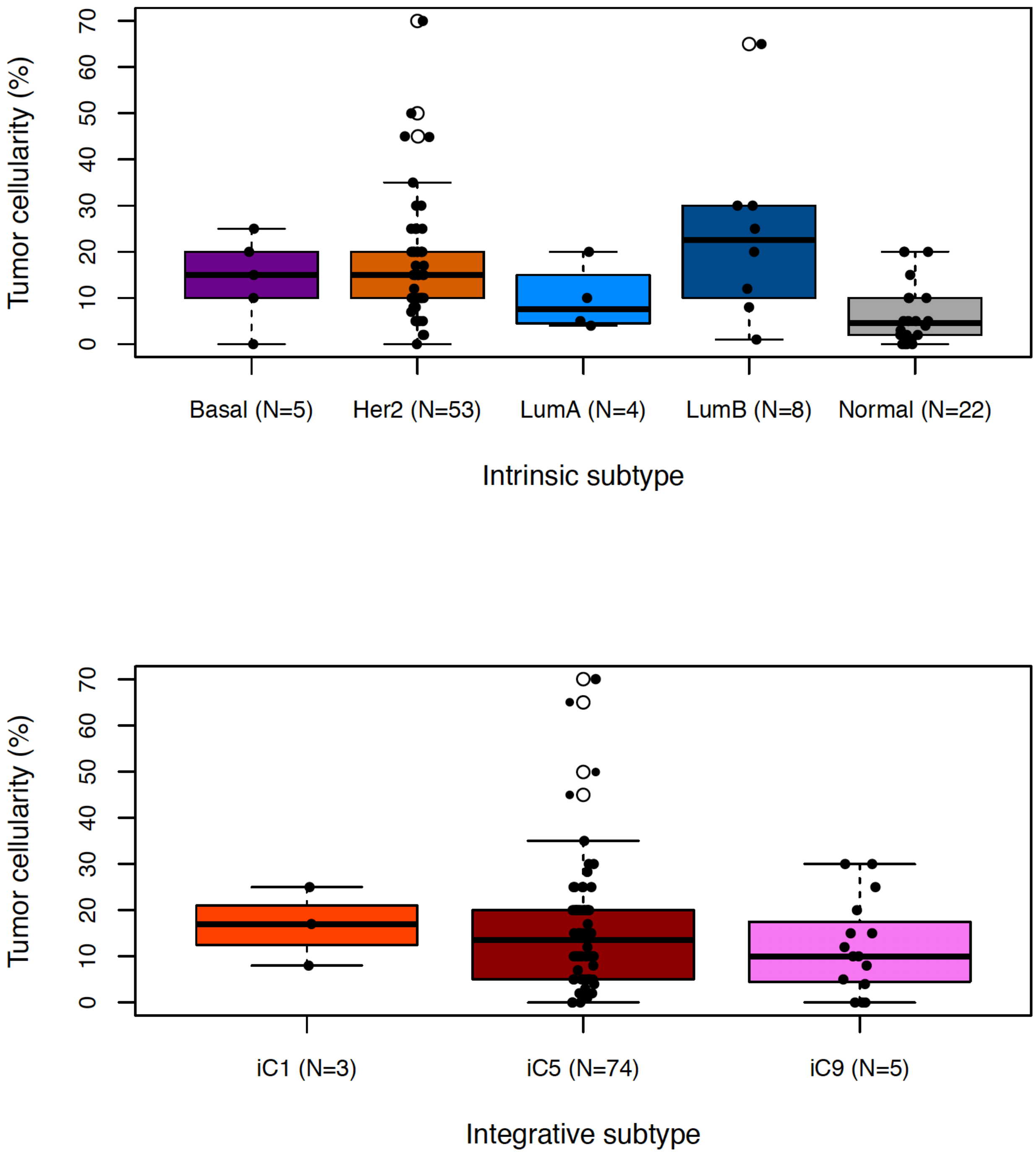

Supplementary Figure 1. Tumor cellularity by intrinsic and integrative subtype. Center line is median, box limits are upper and lower quartiles, whiskers are 1.5x the interquartile range, and empty points are outliers.

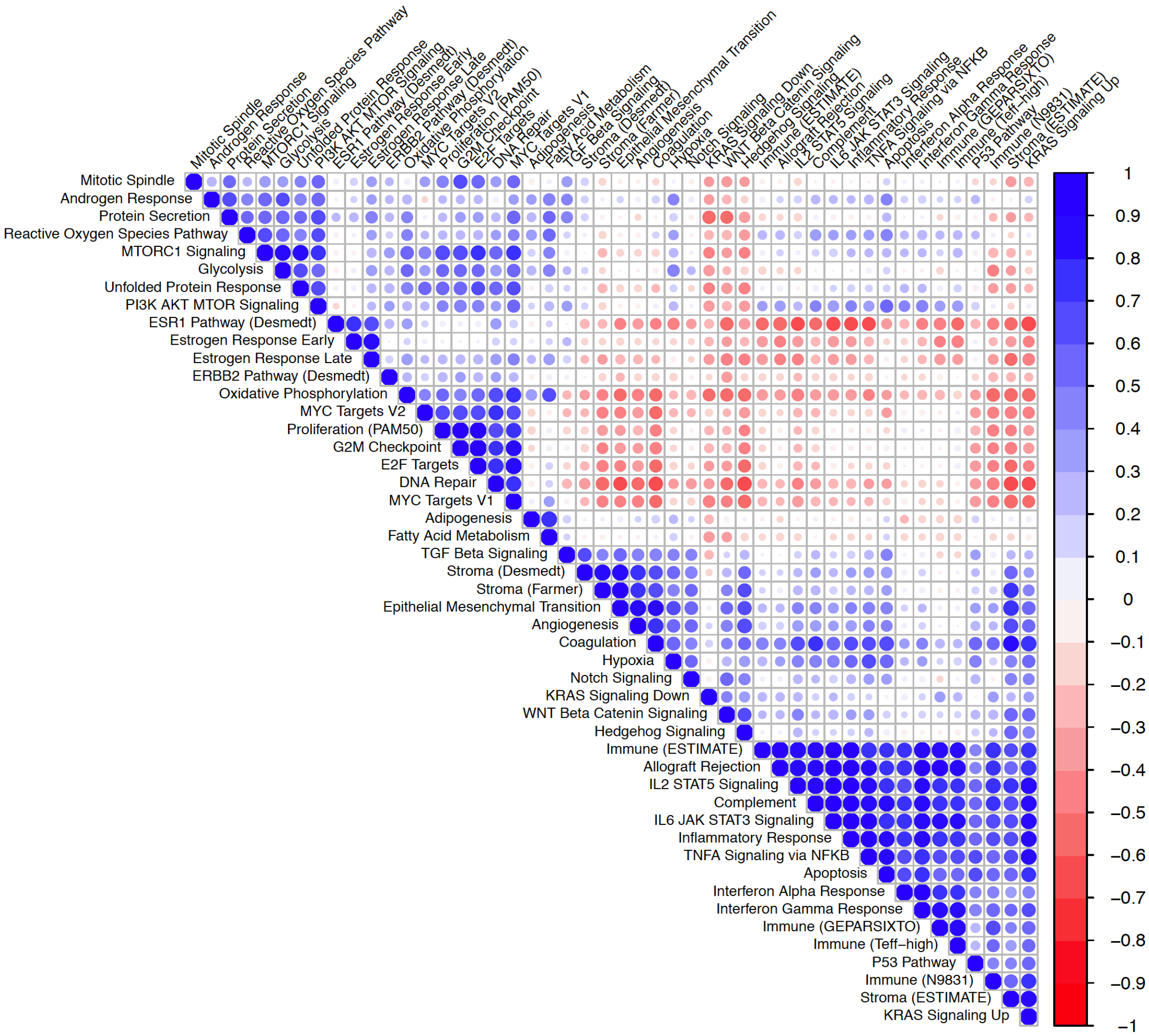

Supplementary Figure 2. Correlation of breast cancer signatures pre-treatment. Pearson correlation coefficient matrix of 48 gene sets in N=110 pre-treatment tumors. Ordering of gene sets is based on hierarchical clustering. Gene sets without labeled source in parentheses are Hallmark Molecular Signatures.

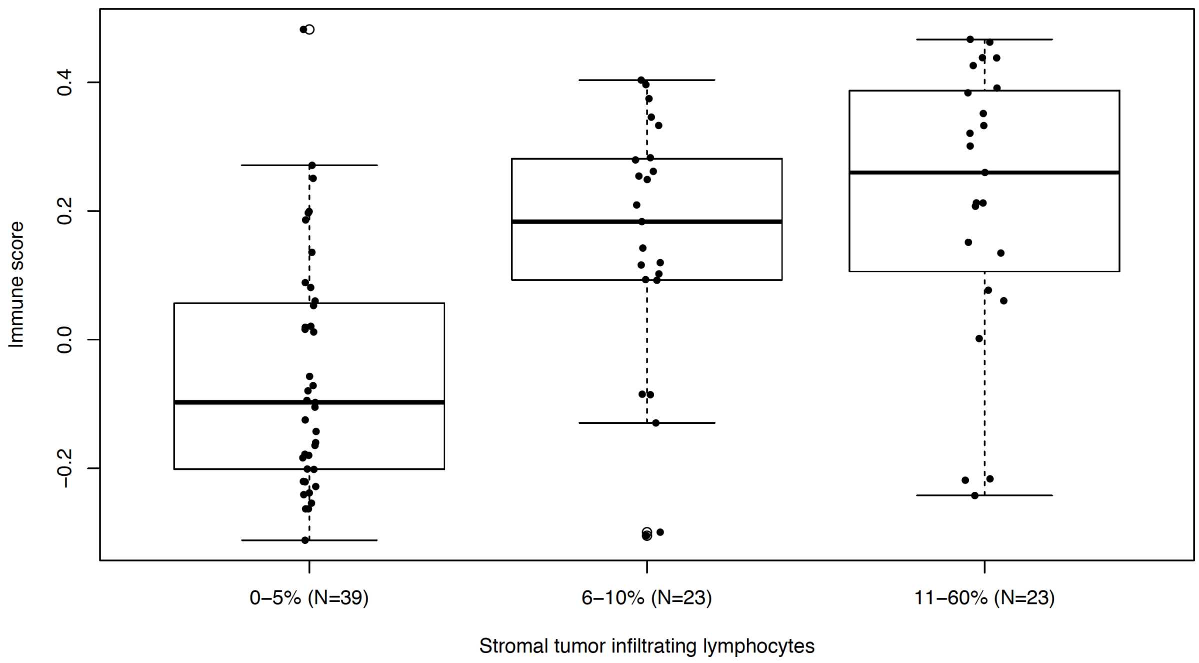

Supplementary Figure 3. Immune scores and stromal TILs pre-treatment. Box plots showing distributions of immune scores (GeparSixto gene set) for different strata of sTILs. Center line is median, box limits are upper and lower quartiles, whiskers are 1.5x the interquartile range, and empty points are outliers. Two-sided *t*-test p-values are p=1.2e-5 comparing 11-60% to 0-5%; p=3.5e-4 comparing 6-10% to 0-5%; and p=0.27 comparing 11-60% to 6-10%.

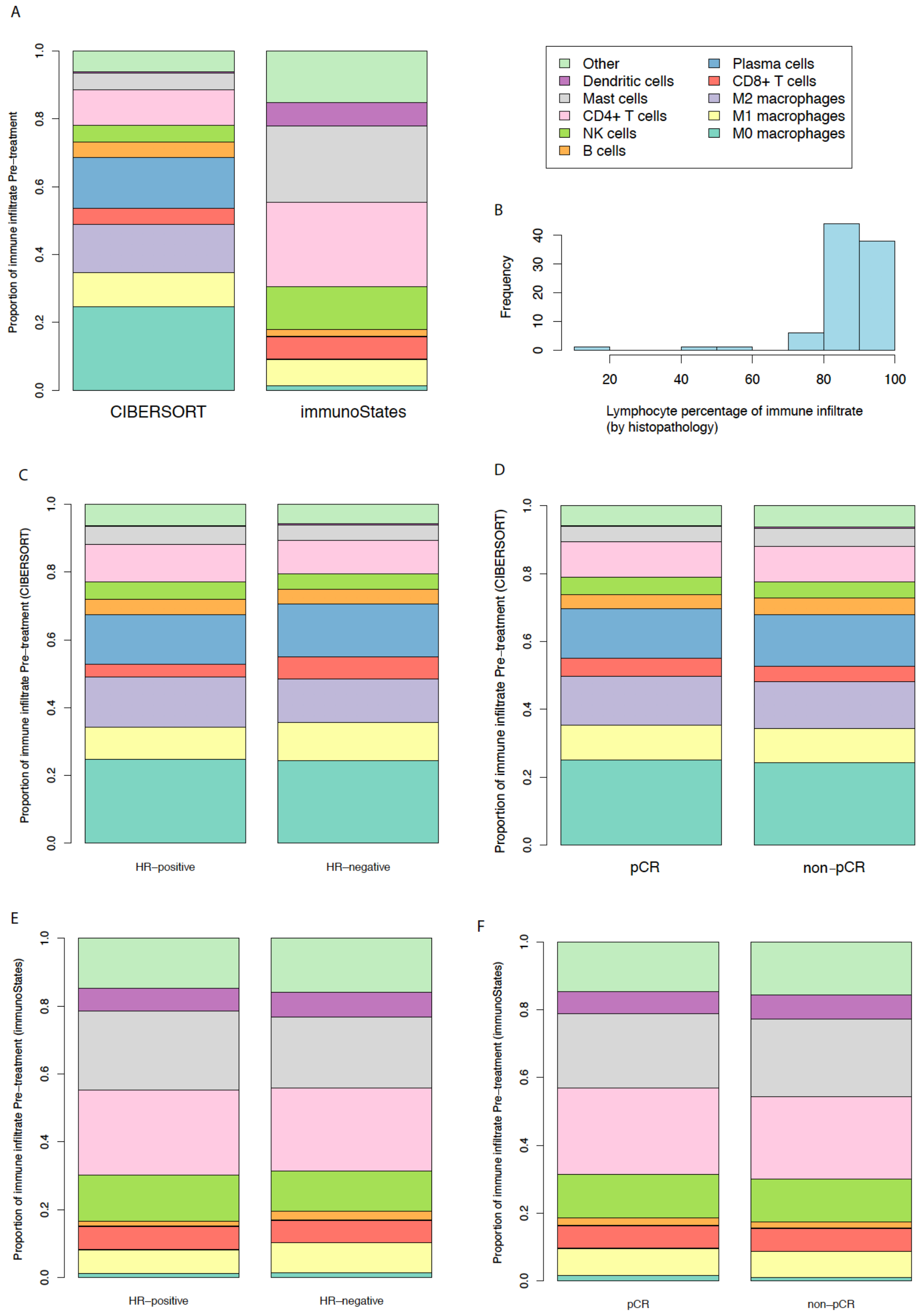
A
Proportion of immune infiltrate Pre−treatment
CIBERSORT
immunoStates
Other
Dendritic cells
Mast cells
CD4+ T cells
NK cells
B cells
Plasma cells
CD8+ T cells
M2 macrophages
M1 macrophages
M0 macrophages
B
Frequency
Lymphocyte percentage of immune infiltrate
(by histopathology)
C
Proportion of immune infiltrate Pre−treatment (CIBERSORT)
HR−positive
HR−negative
D
Proportion of immune infiltrate Pre−treatment (CIBERSORT)
pCR
non−pCR
E
Proportion of immune infiltrate Pre−treatment (immunoStates)
HR−positive
HR−negative
F
Proportion of immune infiltrate Pre−treatment (immunoStates)
pCR
non−pCR

Supplementary Figure 4. Immune infiltration of pre-treatment tumors. a. Stacked bar plots showing the mean proportion of each immune cell subtype as determined by CIBERSORT vs immunoStates. b. Distribution of percentage of inflammatory infiltrate estimated to represent lymphocytes based on histopathology. c. Mean proportion of each immune cell subtype per CIBERSORT by hormone receptor status. d. Mean proportion of each immune cell subtype per CIBERSORT by pathologic complete response status. e. Mean proportion of each immune cell subtype per immunoStates by hormone receptor status. f. Mean proportion of each immune cell subtype per immunoStates by pathologic complete response status. HR = hormone receptor; pCR = pathologic complete response.

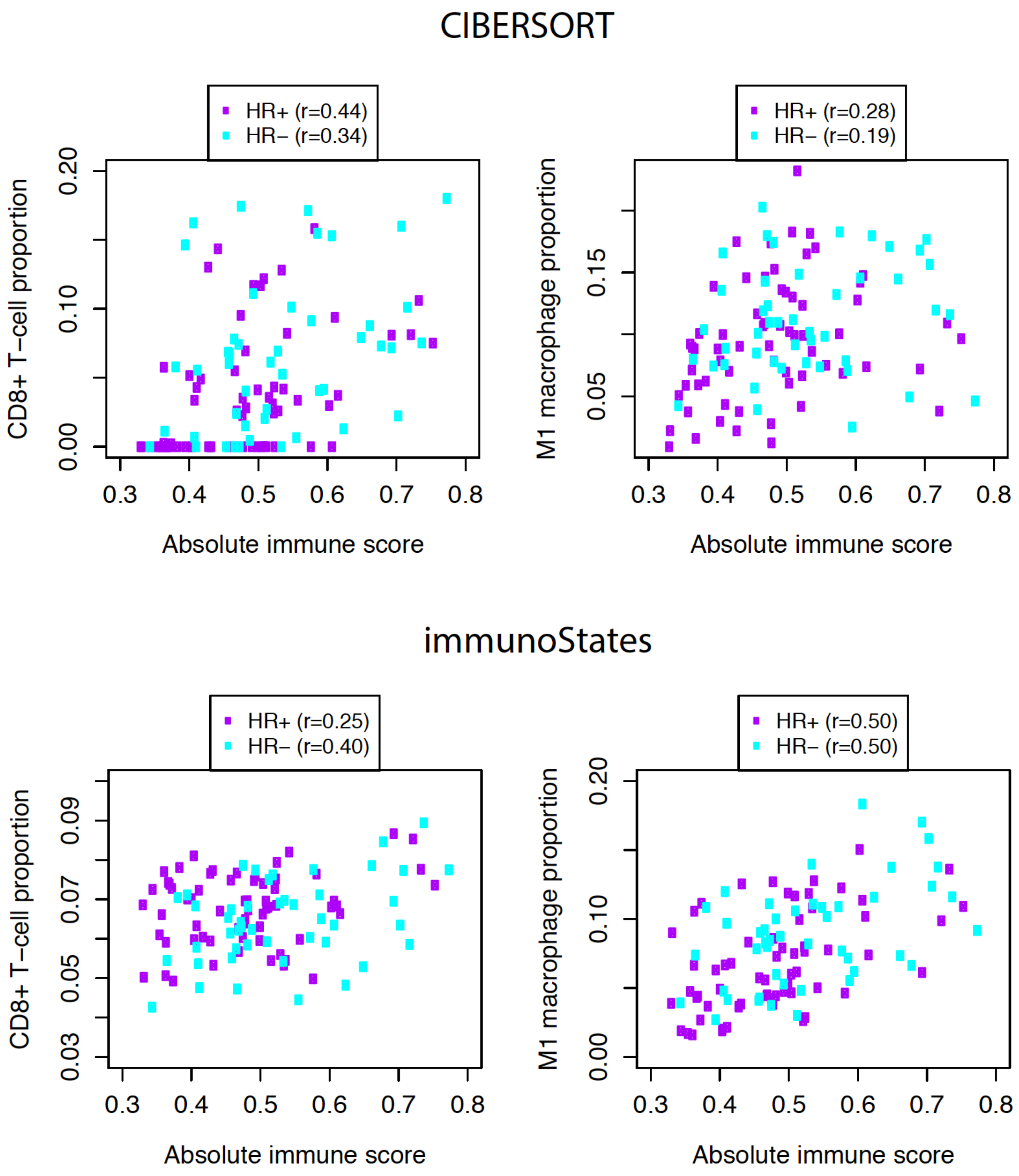

Supplementary Figure 5. Correlation of immune cell subtypes with total immune content. In both CIBERSORT (top) and immunoStates (bottom), tumors with greater absolute immune content (as quantified with CIBERSORT absolute immune score) are estimated to have a higher proportion of CD8+ T-cells and of M1 macrophages. Pearson correlation coefficients are shown by HR subtype. HR = hormone receptor.

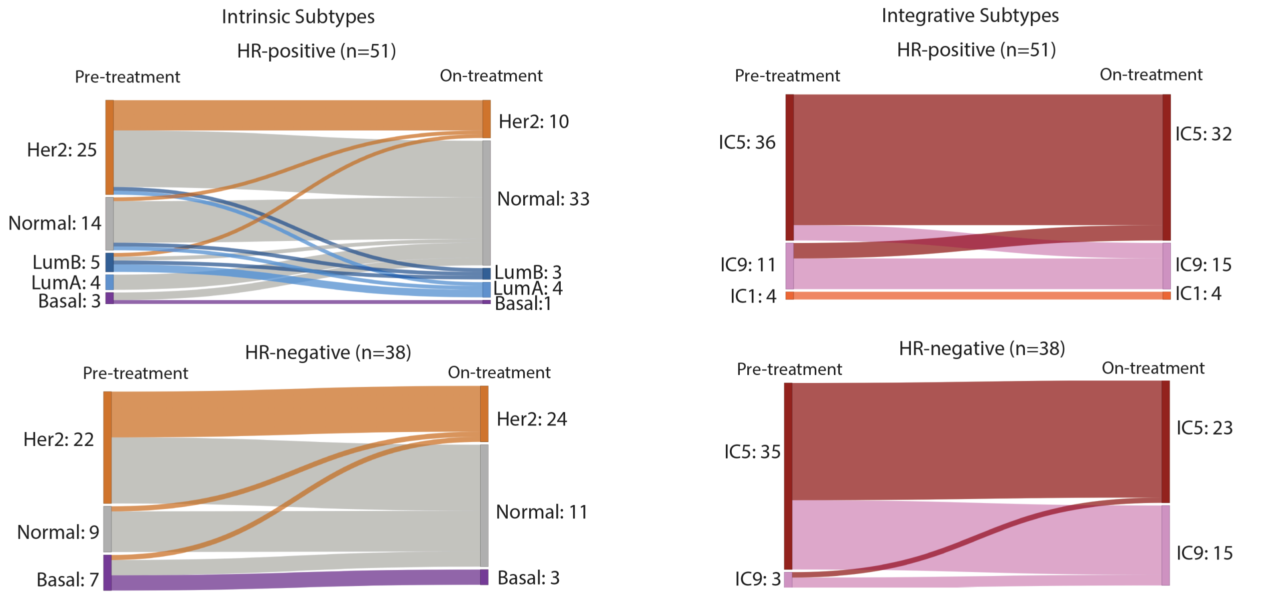

Supplementary Figure 6. Change in tumor subtype after 14-21 days of HER2-targeted therapy by hormone receptor status. Intrinsic subtypes are on the left and integrative subtypes on the right. HR = hormone receptor; IC = integrative cluster.

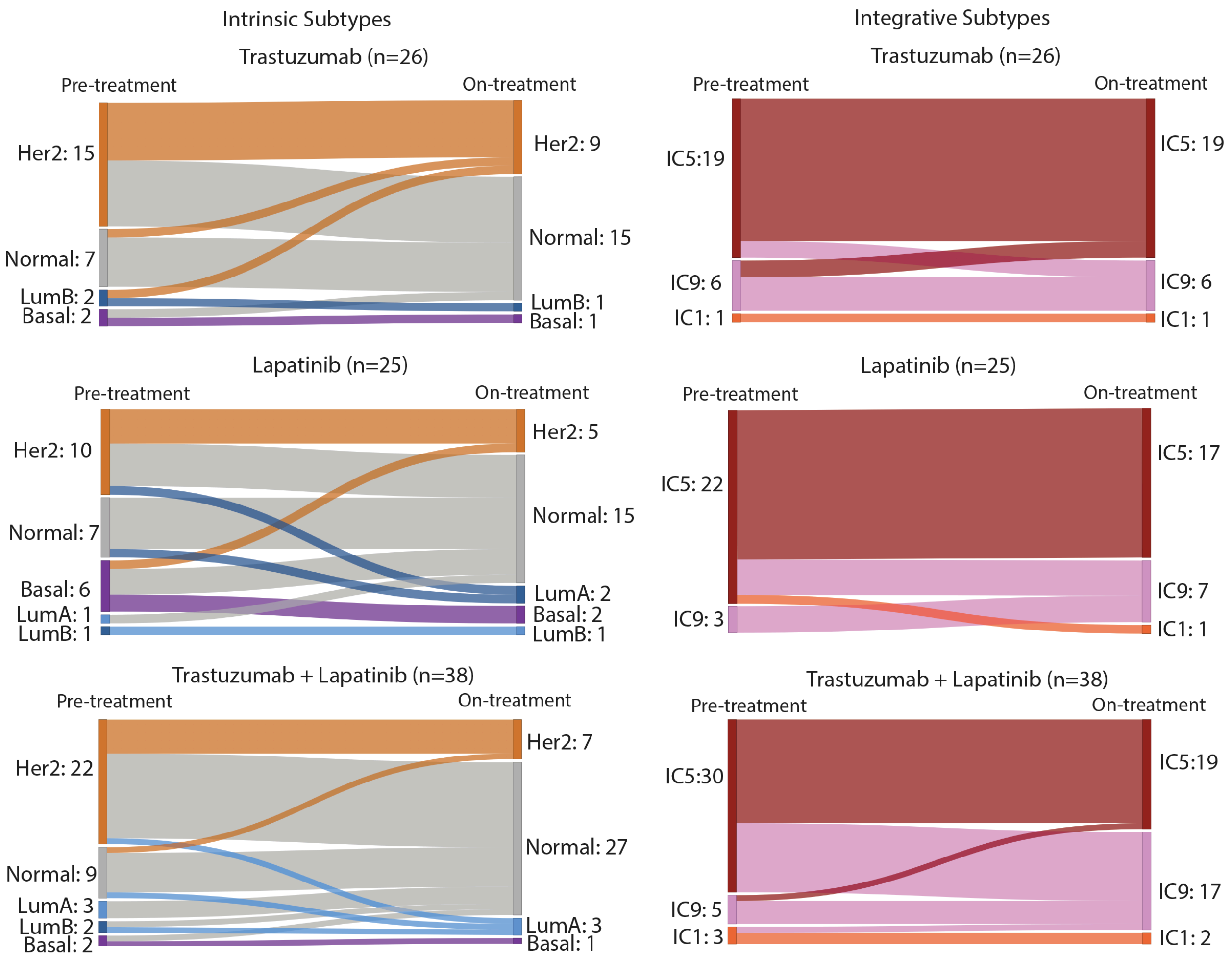

Supplementary Figure 7. Change in tumor subtype after 14-21 days of HER2-targeted therapy by treatment arm. Intrinsic subtypes are on the left and integrative subtypes on the right. IC = integrative cluster.

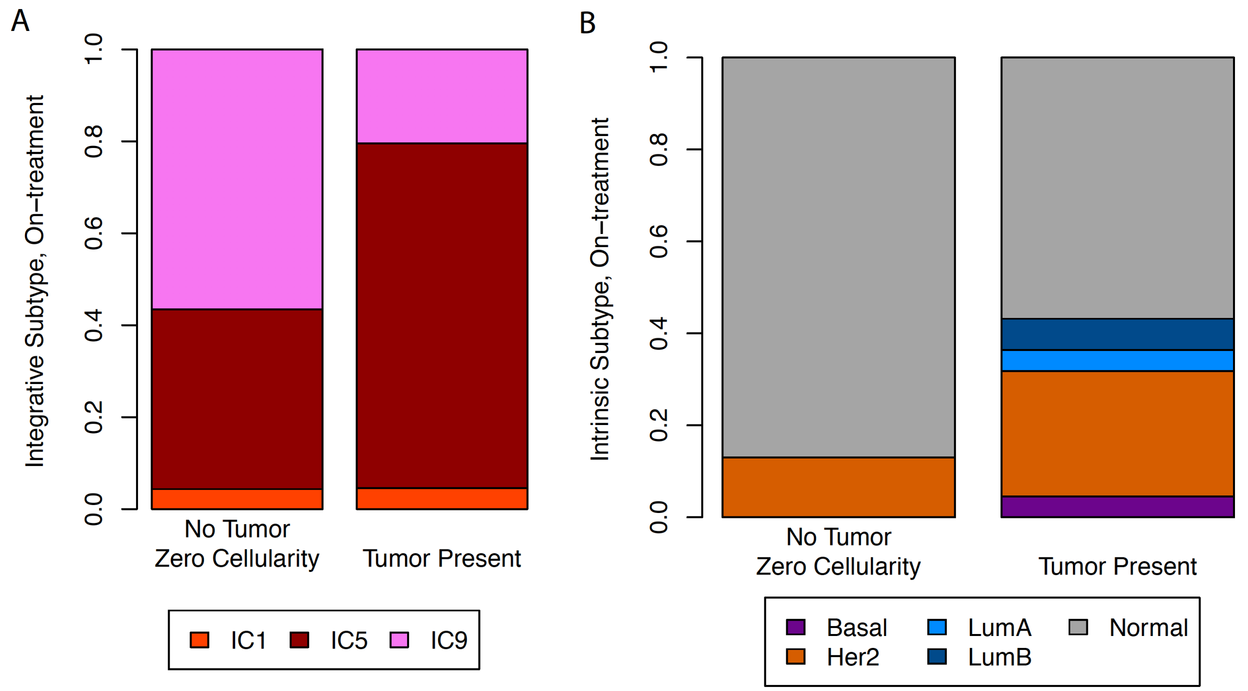

Supplementary Figure 8. Distribution of tumor subtypes after 14-21 days of targeted therapy, comparing biopsies with and without tumor cells identified. IC = integrative cluster.

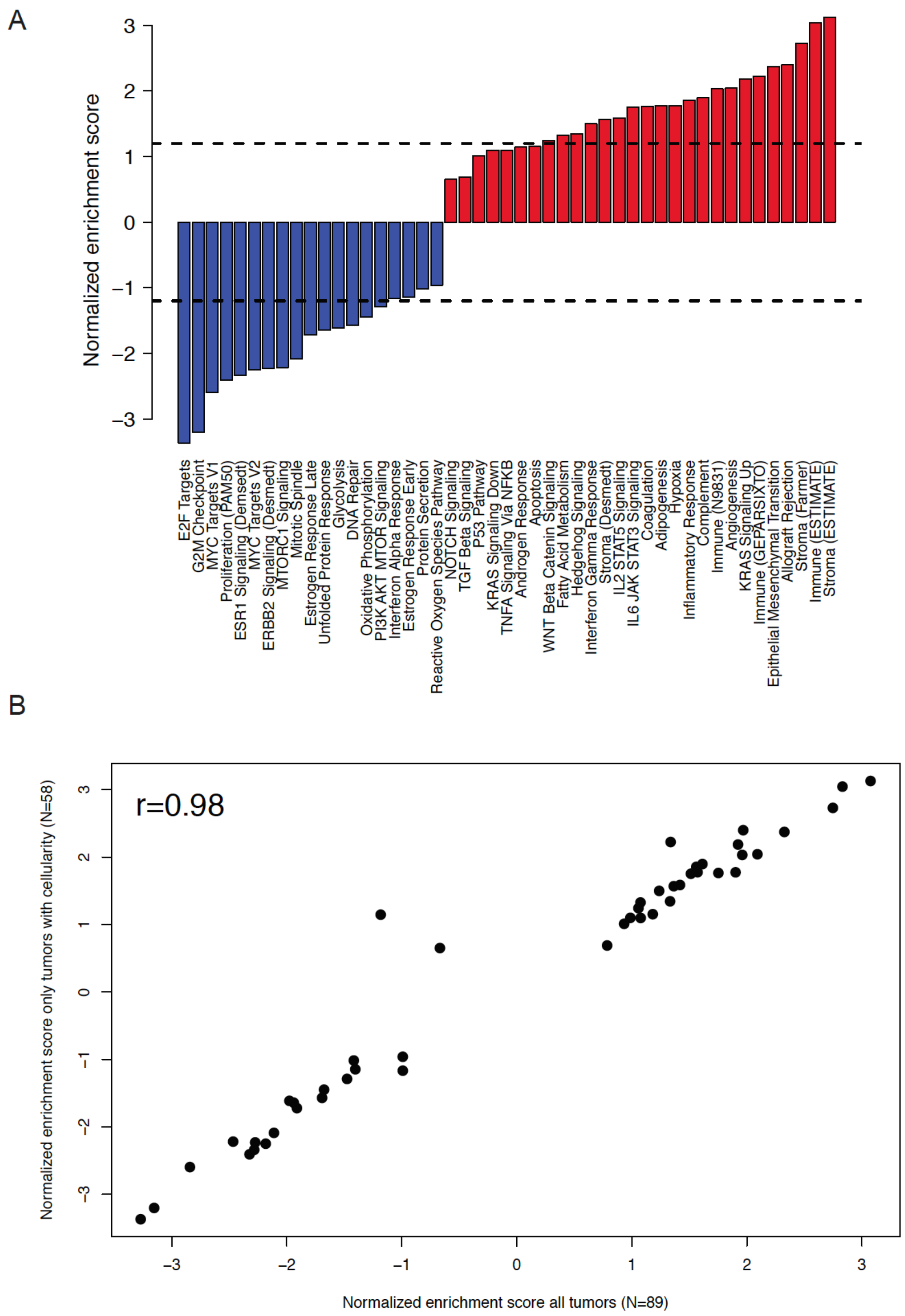

Supplementary Figure 9. Gene set enrichment analysis results for the subset of samples (N=58) with identified tumor present on histopathology 14-21 days after treatment. a. Normalized enrichment scores from gene set enrichment analysis, representing the degree of change of each gene set after 14-21 days of HER2-targeted therapy. Dotted lines separate those with FDR < 0.1. b. Scatter plot showing similarity of normalized enrichment scores in the entire set (x-axis) vs the subset with known tumor (y-axis).

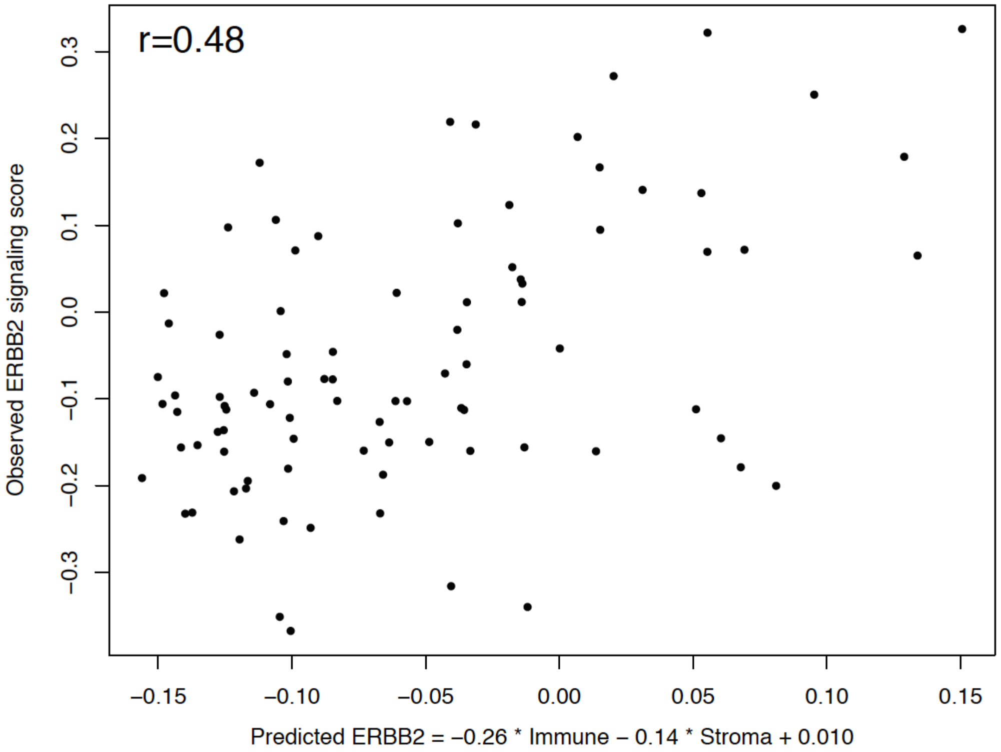

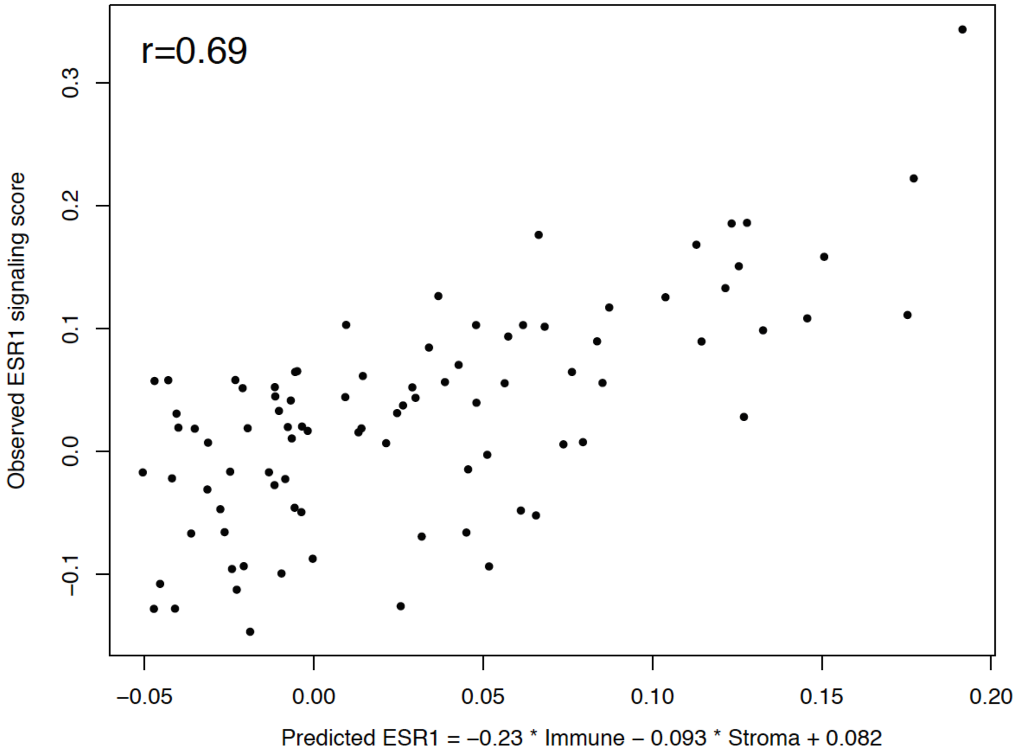

Supplementary Figure 10. Observed ERBB2 (top) and ESR1 (bottom) signaling scores vs predicted scores from immune and stroma scores. Immune score is from ESTIMATE and stroma score is from Farmer et al. Pearson correlation coefficients are shown.

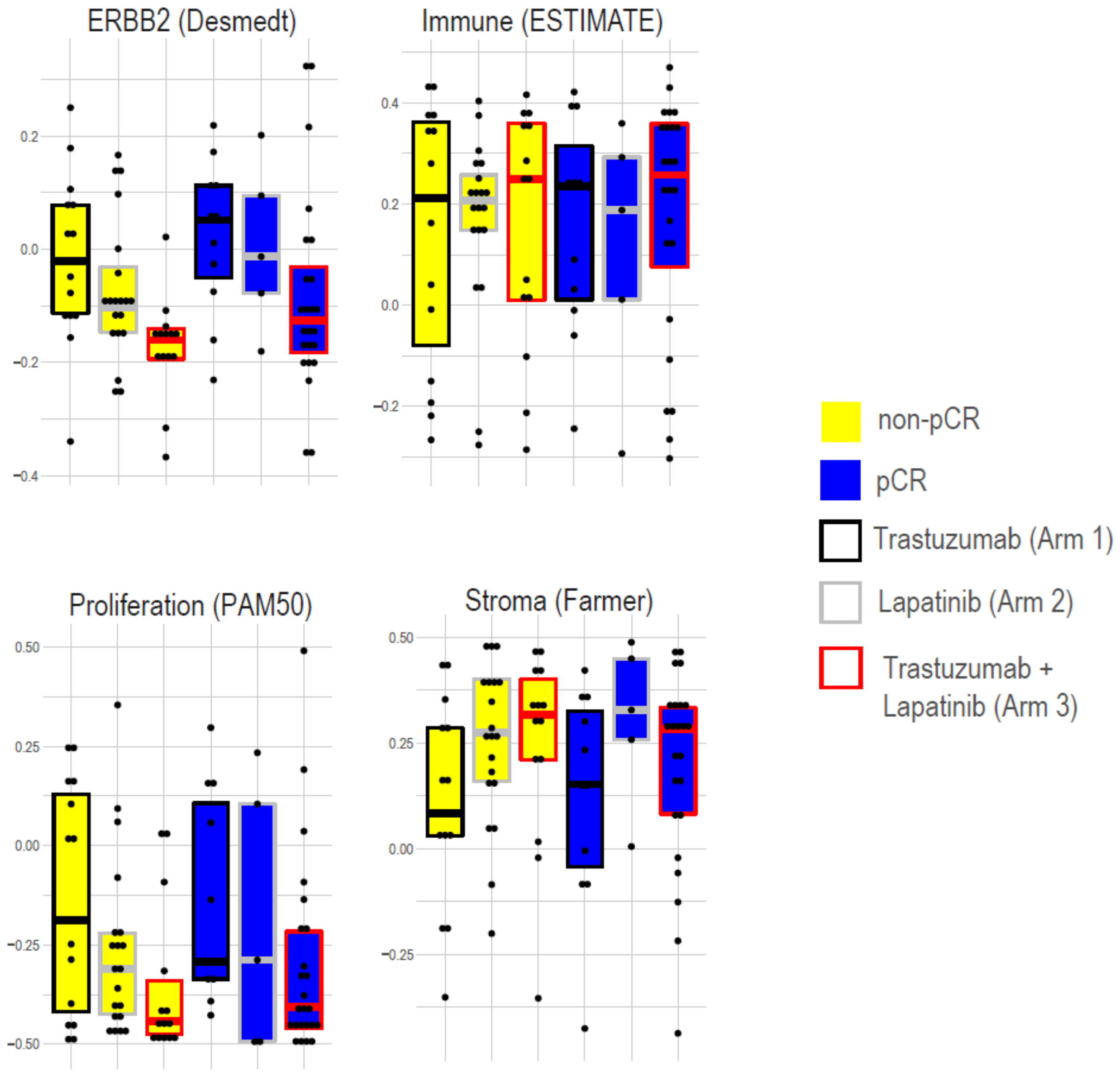

Supplementary Figure 11. Changes in gene sets after 14-21 days of HER2-targeted therapy by treatment arm and pathologic complete response status. Each change is quantified by the mean single-sample gene set enrichment score for the on-treatment tumor compared against its pre-treatment control. Center line is median; box limits are upper and lower quartiles. No gene set change correlated with pCR. Proliferation decreased more with trastuzumab + lapatinib (N=38) than with trastuzumab (N=26) (two-sided *t*-test p=0.0087, FDR-adjusted p=0.078), and stroma increased more with lapatinib (N=25) than with trastuzumab (N=26) (two-sided *t*-test p=0.020, FDR-adjusted p=0.078). pCR = pathologic complete response.

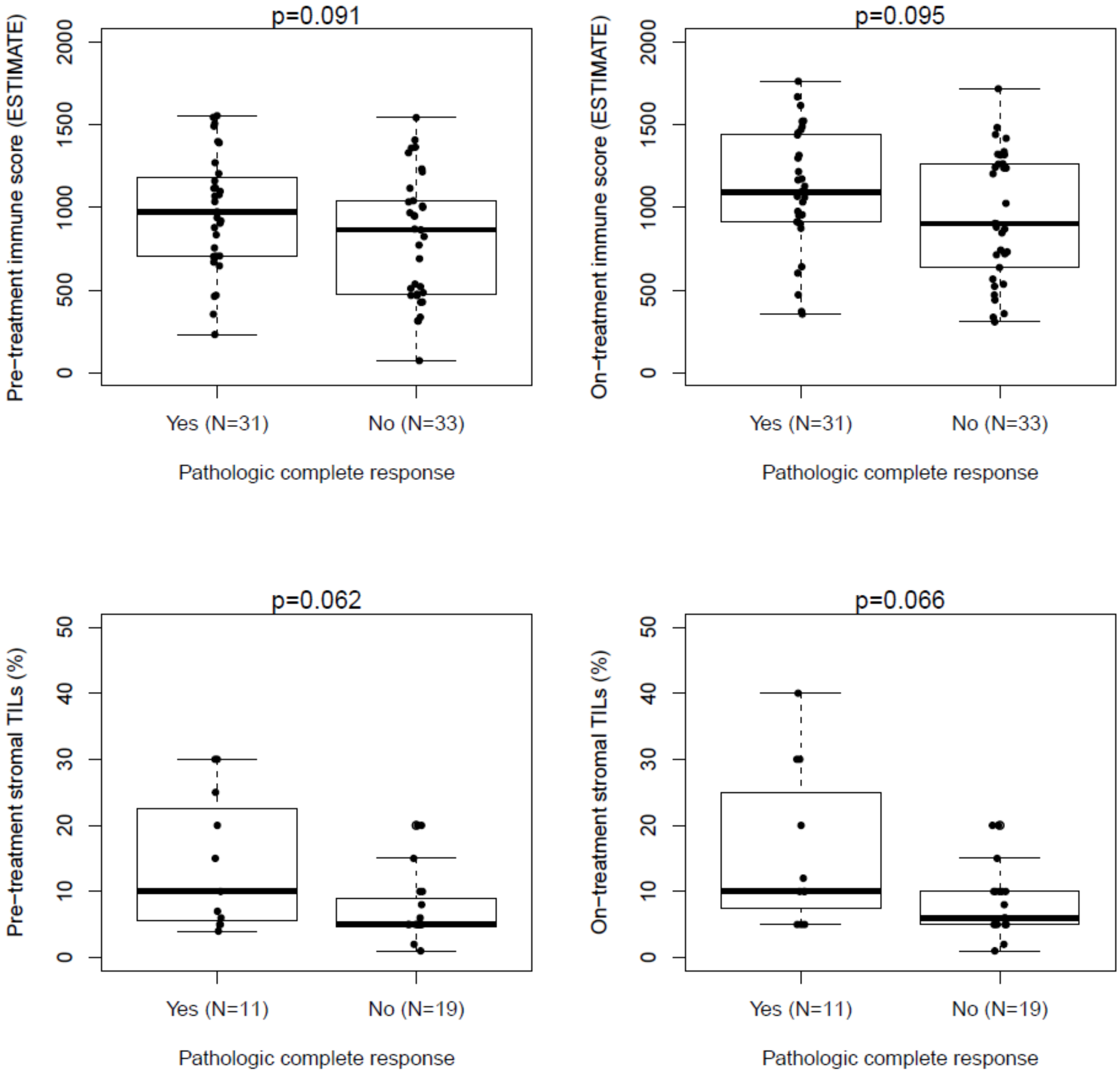

Supplementary Figure 12. Immune infiltration pre-treatment and after 14-21 days of trastuzumab-based HER2-targeted therapy correlate similarly with pathologic complete response (pCR). For immune scores (top), subset of tumors with on-treatment sample available are shown to facilitate comparison. For stromal tumor infiltrating lymphocyte (TIL) percentage (bottom), subset of tumors with on-treatment sample available are shown to facilitate comparison. Lapatinib-only arm excluded given no correlation between immune score or stromal TILs and pCR in this group. Center line is median, box limits are upper and lower quartiles, and whiskers are 1.5x the interquartile range. *P*-values are from two-sided *t*-tests.

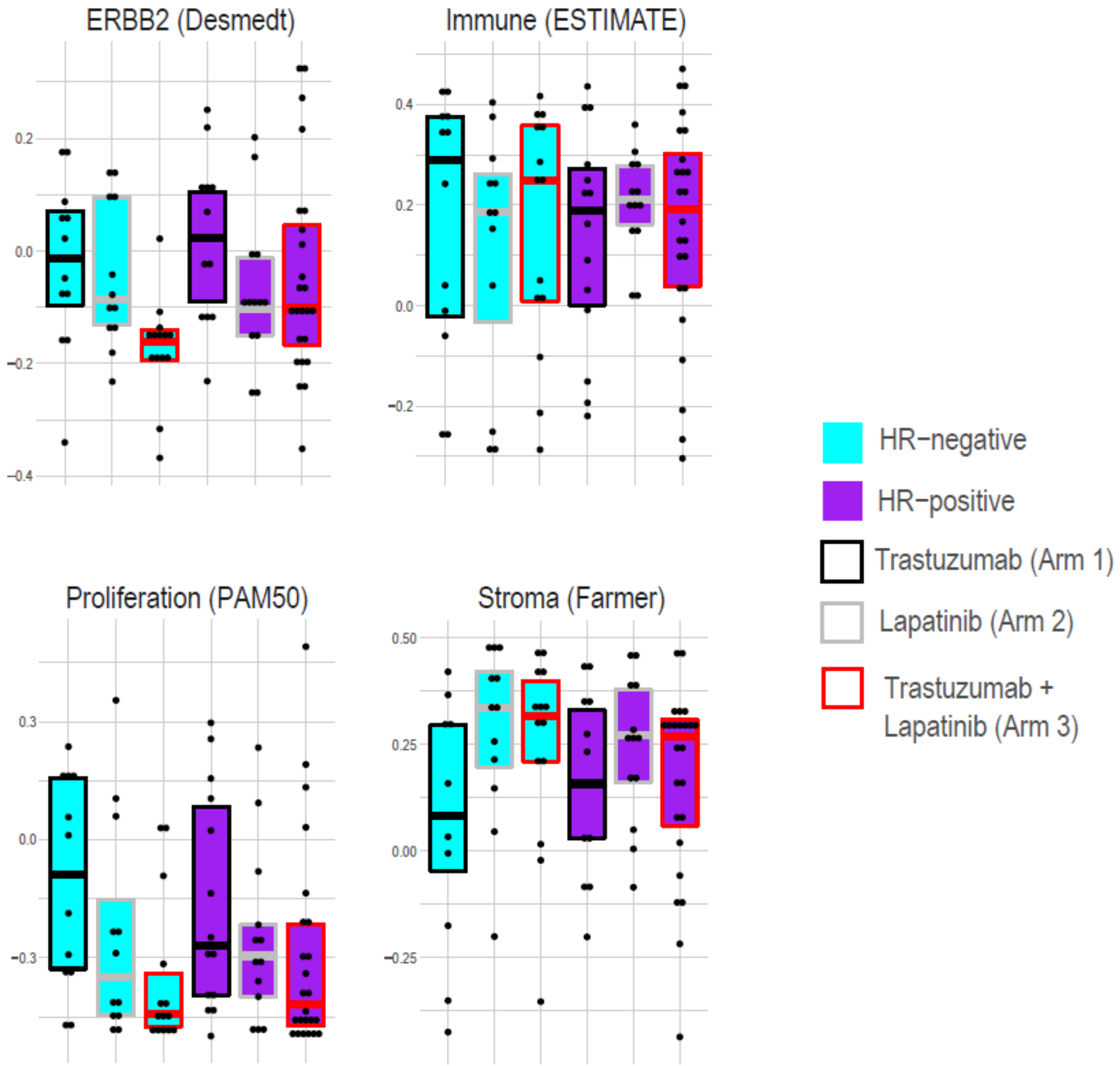

Supplementary Figure 13. Changes in gene sets after 14-21 days of HER2-targeted therapy by treatment arm and pathologic complete response status. Each change is quantified by the mean single-sample gene set enrichment score for the on-treatment tumor compared against its pre-treatment control. Center line is median; box limits are upper and lower quartiles. No gene set change correlated with HR-status. Proliferation decreased more with trastuzumab + lapatinib (N=38) than with trastuzumab (N=26) (two-sided *t*-test p=0.0087, FDR-adjusted p=0.078), and stroma increased more with lapatinib (N=25) than with trastuzumab (N=26) (two-sided *t*-test p=0.020, FDR-adjusted p=0.078). HR = hormone receptor.
