# Supplementary material for: Pathologic and molecular responses to neoadjuvant trastuzumab and/or lapatinib from a phase II randomized trial in HER2-positive breast cancer (TRIO-US B07)
Source: Nat Commun. 2020 Nov 17;11:5824. doi: 10.1038/s41467-020-19494-2 (PMC7673127; doi:10.1038/s41467-020-19494-2)
Supplement: Supplementary file 3 — Reporting Summary [file 41467_2020_19494_MOESM3_ESM.pdf]

nature research

Corresponding author(s): Sara Hurvitz, MD
Christina Curtis, PhD

Last updated by author(s): Oct 6, 2020

Reporting Summary

Nature Research wishes to improve the reproducibility of the work that we publish. This form provides structure for consistency and transparency in reporting. For further information on Nature Research policies, see Authors & Referees and the Editorial Policy Checklist.

Statistics

For all statistical analyses, confirm that the following items are present in the figure legend, table legend, main text, or Methods section.

n/a	Confirmed	
☐	☒	The exact sample size (*n*) for each experimental group/condition, given as a discrete number and unit of measurement
☒	☐	A statement on whether measurements were taken from distinct samples or whether the same sample was measured repeatedly
☐	☒	The statistical test(s) used AND whether they are one- or two-sided<br>*Only common tests should be described solely by name; describe more complex techniques in the Methods section.*
☐	☒	A description of all covariates tested
☐	☒	A description of any assumptions or corrections, such as tests of normality and adjustment for multiple comparisons
☐	☒	A full description of the statistical parameters including central tendency (e.g. means) or other basic estimates (e.g. regression coefficient) AND variation (e.g. standard deviation) or associated estimates of uncertainty (e.g. confidence intervals)
☐	☒	For null hypothesis testing, the test statistic (e.g. *F*, *t*, *r*) with confidence intervals, effect sizes, degrees of freedom and *P* value noted<br>*Give P values as exact values whenever suitable.*
☒	☐	For Bayesian analysis, information on the choice of priors and Markov chain Monte Carlo settings
☒	☐	For hierarchical and complex designs, identification of the appropriate level for tests and full reporting of outcomes
☐	☒	Estimates of effect sizes (e.g. Cohen's *d*, Pearson's *r*), indicating how they were calculated

Our web collection on statistics for biologists contains articles on many of the points above.

Software and code

Policy information about availability of computer code

Data collection	Agilent microarray expression data were extracted using Agilent Feature Extraction Software versions 10.7 and 11.0. IHC images were digitized on the Aperio Digital Pathology Slide Scanner (Leica Biosystems). The Aperio ImageScope software (Leica Biosystems) (version 12.3.2.8013) was used for image visualization and acquisition.
Data analysis	The following software was used for data analysis:<br>R versions 3.5.1 and 3.6.1 with additional packages limma (3.28.21), sva (3.20.0), AIMS (1.4.0), genefu (2.16.0), MetaIntegrator (2.1.1), GSVA (1.32.0), GSEABase (1.46.0), ESTIMATE (1.0.13)<br>GSEA version 4.0.2<br>CIBERSORT version 1.06

For manuscripts utilizing custom algorithms or software that are central to the research but not yet described in published literature, software must be made available to editors/reviewers. We strongly encourage code deposition in a community repository (e.g. GitHub). See the Nature Research guidelines for submitting code & software for further information.

Data

Policy information about availability of data

All manuscripts must include a data availability statement. This statement should provide the following information, where applicable:

- Accession codes, unique identifiers, or web links for publicly available datasets
- A list of figures that have associated raw data
- A description of any restrictions on data availability

Raw expression data are deposited in GEO (GSE130788). The full trial protocol and processed data associated with the manuscript are available online at github.com/cancersysbio/TRIOB07/. The processed data files include: preTreatment.txt (Figures 2, S1-S5, S13, 4C-D), onTreatment.txt (Figures 3A, 3C-F, S6-S9, S11-S14, 4C-D), postTreatment.txt (4C-D), and GSEA.txt (Figures 3B, S10, 4A-B).

Field-specific reporting

Please select the one below that is the best fit for your research. If you are not sure, read the appropriate sections before making your selection.

☒ Life sciences ☐ Behavioural & social sciences ☐ Ecological, evolutionary & environmental sciences

For a reference copy of the document with all sections, see nature.com/documents/nr-reporting-summary-flat.pdf

Life sciences study design

All studies must disclose on these points even when the disclosure is negative.

Sample size	The primary objective was to estimate the pCR rate in Arm 3 (the combination therapy arm with trastuzumab and lapatinib). The aim was to detect an absolute 20% difference between the pCR rate with the treatment in that arm (with hypothesized 60% pCR rate) and the historical control pCR rate (of ~40%). With a nominal one-sided 0.05 significance and 90% power, using the exact binomial method, a sample size of 56 participants in each arm was estimated to be required. Ultimately, the trial accrued 58 participants to that arm, 34 participants to Arm 1, and 36 participants to Arm 2. Secondary objectives, including molecular analyses, were performed in an exploratory fashion with the sample sizes available in the trial.
Data exclusions	Exclusion criteria for participation in the clinical trial included prior exposure to chemotherapy, radiation, or endocrine therapy for currently diagnosed invasive or non-invasive breast cancer, any prior radiation therapy to ipsilateral breast or chest wall, history of any other malignancy within the past 5 years (except non-melanoma skin cancer or carcinoma-in-situ of the cervix), pre-existing motor or sensory neuropathy of grade >2, pre-existing cardiac disease, gastrointestinal condition causing chronic diarrhea requiring active therapy, concurrent infection requiring parenteral antibiotics, metastatic breast cancer, current treatment with ovarian hormonal replacement therapy, or current treatment with any selective estrogen receptor modulators. Pregnant or lactating women were excluded and contraception was required for females of childbearing potential.<br><br>Two of the initially 130 enrolled participants withdrew prior to starting any treatment, and were excluded from the primary intention-to-treat analysis. The 25 participants who came off their assigned study treatment prior to surgery and 1 participant who did not complete surgery were included in the primary intention-to-treat analysis, and excluded in the pre-specified evaluable population analysis.<br><br>Gene expression data, immunohistochemistry, and histopathology were available for a subset of participants (because of lack of tissue, insufficient RNA quantity or quality from tissue, or lack of matched pathology report). No collected data were excluded from the molecular analyses.
[illegible]eplication	This was a study of N=130 participants and provides estimates of pCR rates across three different arms. Other studies have also examined the question of differences in pCR rates with use of lapatinib, trastuzumab, or the combination, and we discuss similarities and differences in the Discussion. The gene expression results, which were not based on pre-specified hypotheses, will require validation, which we intend to pursue but which are outside the scope of the present effort, and will necessitate major prospective efforts.
Randomization	Participants were stratified based on baseline tumor size (<3 cm and >3 cm) and hormone receptor status (HR+ vs HR-). A random permuted block design was utilized for randomization, with the block size varied between 3 and 6 at random. The study statistician generated the random allocation sequence sequence.
Blinding	Placebo was not used for trastuzumab nor lapatinib, as this would have substantially increased the burden on participants and cost of the trial. Pathologists were blinded to arm and clinical outcome when interpreting the histopathologic and immunohistochemical data.

Reporting for specific materials, systems and methods

We require information from authors about some types of materials, experimental systems and methods used in many studies. Here, indicate whether each material, system or method listed is relevant to your study. If you are not sure if a list item applies to your research, read the appropriate section before selecting a response.

Materials & experimental systems

n/a	Involved in the study	
☐	☒	Antibodies
☒	☐	Eukaryotic cell lines
☒	☐	Palaeontology
☒	☐	Animals and other organisms
☐	☒	Human research participants
☐	☒	Clinical data

Methods

n/a	Involved in the study	
☒	☐	ChIP-seq
☒	☐	Flow cytometry
☒	☐	MRI-based neuroimaging

Antibodies

[illegible]ntibodies used	Immunohistochemistry for Ki67 (Agilent clone MIB-1; M7240; dilution 1:200) and ER-alpha (Agilent clone SP1; M3634; dilution 1:200)
Validation	The above monoclonal mouse anti-human Ki-67 antigen is commercially available from Agilent with intended application immunohistochemistry. It is a diagnostic marker validated on human diagnostic tissue.

The above monoclonal rabbit anti-human ER-alpha antigen was commercially available from Agilent with intended application semi-quantitative immunohistochemistry in formalin-fixed, paraffin-embedded tissue sections of human breast cancer. It is a diagnostic marker validated on human diagnostic tissue.

Human research participants

Policy information about studies involving human research participants

Population characteristics

Of enrolled participants, median age was 48 (range 27-78). Tumors were all HER2-positive; 44% were hormone receptor-negative and 56% were hormone receptor-positive. 5% of tumors were clinical anatomic stage I, 67% were clinical anatomic stage II, and 28% were clinical anatomic stage III. No prior chemotherapy, radiotherapy, or endocrine therapy for currently diagnosed breast cancer was allowed upon entry into the study.

Recruitment

Participants were recruited at 13 oncology centers in the United States through the Translational Research in Oncology network. In an open-label clinical trial, there is the potential for selection bias as participants may elect to drop out of randomized to an arm they perceive to be associated with less benefit. This could cause imbalance in measured/unmeasured baseline characteristics of patients in the treatment arms. Two patients dropped out of this study prior to receiving therapy; one in the control arm (TCH) due to discovery of metastatic disease and one in the TCL arm due to withdrawal of consent. These two dropouts were not likely to have caused an imbalance in the patients. Another potential source of bias is early discontinuation due to toxicity of the regimen. This could affect the pCR rate as well as correlative biomarker analyses. In this study, the use of lapatinib in the experimental arms was associated with increased toxicity, leading to a decreased rate of trial completion, as discussed in the manuscript.

Ethics oversight

The following institutional review boards approved the study protocol: UCLA, Olive View, and Western. The Stanford University institutional review board also approved the gene expression analyses.

Note that full information on the approval of the study protocol must also be provided in the manuscript.

Clinical data

Policy information about clinical studies

All manuscripts should comply with the ICMJE guidelines for publication of clinical research and a completed CONSORT checklist must be included with all submissions.

Clinical trial registration

NCT00769470

Study protocol

TRIO TORI B07 Amendment#3 3-19-13, available on github.com/cancersysbio/TRIOB07

Data collection

Participants were treated and data collected at the University of California, Los Angeles (UCLA) (including satellite clinics at Santa Monica, Valencia, UCLA Westlake, and Pasadena) and twelve additional United States sites through the Translational Research In Oncology (TRIO)-US network (Bakersfield, Fullerton, Redondo Beach, Inland Valleys, Santa Maria, Orlando (Florida), Santa Barbara [2 sites], Las Vegas, Olive View, Hollywood (Florida), and San Luis Obispo). Participants enrolled from December 2008 to December 2012. Primary completion date of the study was November 2013.

Outcomes

The primary objective was to investigate the clinical efficacy of docetaxel/carboplatin in combination with trastuzumab, lapatinib, or trastuzumab/lapatinib by estimating the pathologic complete response rate in the breast and axilla.

Secondary objectives included estimation of the molecular effects of lapatinib alone, trastuzumab alone, and lapatinib combined with trastuzumab by assessing changes in gene expression using serial gene microarray analysis; assessment for gene expression changes that might be correlated with or predict pCR and clinical response to lapatinib and/or trastuzumab; evaluation of the safety and tolerability of the three treatment arms; evaluation of the clinical efficacy of the three treatment arms by estimating the clinical objective response rate; and estimation of the rate of congestive heart failure or drop in left ventricular ejection fraction (>10% points from baseline and below lower limit of normal) in each of the three treatment arms).
